# Screening high-risk individuals for primary gastric adenocarcinoma: evaluating progression-free survival probability score in the presence and absence of Rictor expression after gastrectomy

**DOI:** 10.3389/fonc.2024.1382818

**Published:** 2024-11-11

**Authors:** Jian Wang, Yifan Li, Sujiao Liang

**Affiliations:** ^1^ Hepatobiliary, Pancreatic and Gastrointestinal Surgery, Shanxi Hospital Affiliated to Carcinoma Hospital, Chinese Academy of Medical Sciences, Shanxi Province Carcinoma Hospital, Carcinoma Hospital Affiliated to Shanxi Medical University, Taiyuan, Shanxi, China; ^2^ Department of Pharmacy, Shanxi Province Cancer Hospital, Shanxi Hospital Affiliated to Cancer Hospital, Chinese Academy of Medical Sciences, Cancer Hospital Affiliated to Shanxi Medical University, Taiyuan, Shanxi, China

**Keywords:** gastric adenocarcinoma, Rictor, progression-free survival, nomogram, risk stratification

## Abstract

**Objective:**

Developing nomogram-based risk stratification model to determine 3-year and 5-year progression-free survival (PFS) and to identify high-risk patients with gastric adenocarcinoma based on different Rictor statuses.

**Methods:**

1366 individuals who underwent radical gastric surgery to treat gastric adenocarcinoma at Shanxi Cancer Hospital from May 2002 to December 2020 were analyzed. Cox regression analysis was employed to create the nomograms. The nomograms’ performance was assessed using C-index, time receiver operating characteristic (t-ROC) curves, calibration curves, and decision curve analysis (DCA) curves in training and validation cohorts. Subsequently, patients were categorized into high-risk and low-risk groups based on the nomogram’s risk scores.

**Results:**

The Rictor (-) nomogram for predicting PFS included variables such as age, number of positive lymph nodes, vascular invasion, maximum diameter of the tumor, omentum metastasis, and expression of MSH2. In the internal validation, the C-index of the Rictor (-) nomogram was 0.760 (95%CI: 0.720-0.799), which was superior to the C-index of the American Joint Committee on Cancer (AJCC) 8th edition TNM staging (0.683, 95%CI: 0.646-0.721). Similarly, the Rictor (+) nomogram for predicting PFS included variables such as gender, age, pT stage, number of positive lymph nodes, neural invasion, maximum diameter of the tumor, omentum metastasis, Clavien-Dindo classification for complications, and CGA expression. The C-index of the Rictor (+) nomogram was 0.795 (95%CI: 0.764-0.825), which outperformed the C-index of the AJCC 8th edition TNM staging (0.693, 95%CI: 0.662-0.723). The calibration curves, t-ROC curves, and decision curve analysis for both nomogram models demonstrated their excellent prediction ability.

**Conclusion:**

This study presents the first risk stratification for Rictor status in gastric adenocarcinoma. Our model identifies low-risk patients who may not require additional postoperative treatment, while high-risk patients should consider targeted therapies that specifically target Rictor-positive indicators.

## Introduction

Gastric cancer, one of the most common types of malignant tumors around the world, ranks fifth in terms of prevalence. Alarmingly, it is also the third leading cause of death among these individuals (1). Although significant progress has been made in standardized D2 lymphadenectomy and subsequent adjuvant chemotherapy, ultimately leading to enhancements in the overall survival (OS) rates of gastric cancer patients (2–4), long-term survival outcomes remain disappointingly unsatisfactory. Research results indicate the urgent requirement for novel targeted drugs to enhance the survival outcomes for patients suffering from advanced or recurring diseases (5). Specifically, molecular therapies aimed at human epidermal growth factor receptor-2 (HER2) and vascular endothelial growth factor receptor (VEGFR) have garnered considerable attention within gastric cancer treatment and have successfully been applied in clinical settings (6, 7). Furthermore, the potential therapeutic targeting of the mammalian target of rapamycin (mTOR) and the utilization of rapamycin inhibitors are currently under investigation, holding promise as an alternative avenue for treatment. In this study, the positive expression rate of Rictor protein in gastric adenocarcinoma was significantly higher (49.8%) than in normal adjacent tissues. In Wang et al.’s study (8), they found that the positive rate of Rictor was 51.6% (129/150) in gastric cancer tissues. Their multivariate analyses revealed that Rictor was an independent unfavorable predictor for overall survival. Additionally, patients with upregulated Rictor in the primary tumor and lymph node metastases had the worst prognosis. This aligns with similar results from another study, which also found that elevated Rictor expression is associated with tumor progression and poor prognosis in patients with gastric cancer (9).

However, it is essential to note that the survival of patients varied widely, showing significant discrepancies between them. As a result, we decided to initiate risk stratification after Rictor expression based on their prediction model. We aim to develop nomograms model that can accurately predict the 3-year and 5-year progression-free survival (PFS) rates for Rictor protein-positive and negative cases, respectively. This model will allow for risk stratification, enabling us to identify patients who would benefit from targeted therapy for Rictor protein-positive cases and those who should avoid unnecessary overtreatment. We aim to provide precise treatment guidance for postoperative gastric adenocarcinoma patients by adopting a precision medicine approach. Ultimately, our goal is to achieve individualized treatment strategies and enhance the effectiveness of personalized therapy.

## Methods

### Data collection

For the study, the records of 1366 individuals who underwent radical gastric surgery to treat gastric adenocarcinoma at Shanxi Cancer Hospital from May 2002 to December 2020 were analyzed, and samples were obtained and preserved for Rictor protein determination. Among these individuals, 676 patients with gastric adenocarcinoma were identified as Rictor positive. The first group with Rictor negativity of 690 patients was randomly assigned to two groups in a ratio of 7:3. The group comprised 486 cases and served as the training cohort. In contrast, the remaining 204 instances formed the validation cohort. Similarly, 676 gastric adenocarcinoma patients with Rictor positivity were carefully selected and divided into two groups in a similar ratio. The training cohort included 496 cases, while the validation cohort comprised 180 cases. This rigorous process ensured a diverse and representative sample for the study.

**Figure 1:**
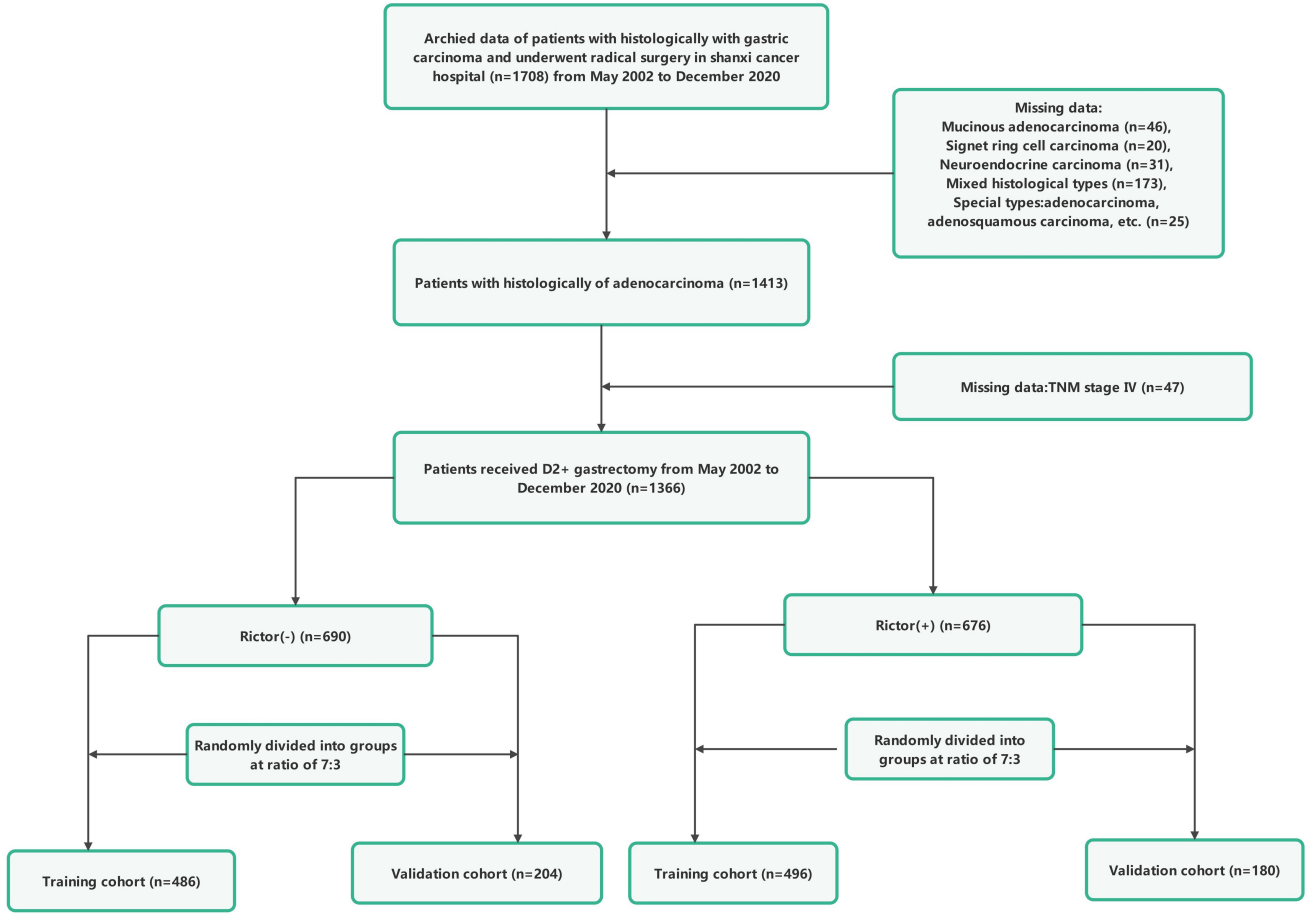
The flowchart of study population enrolment in the training and validation cohort of gastric cancer.

**Figure 2:**
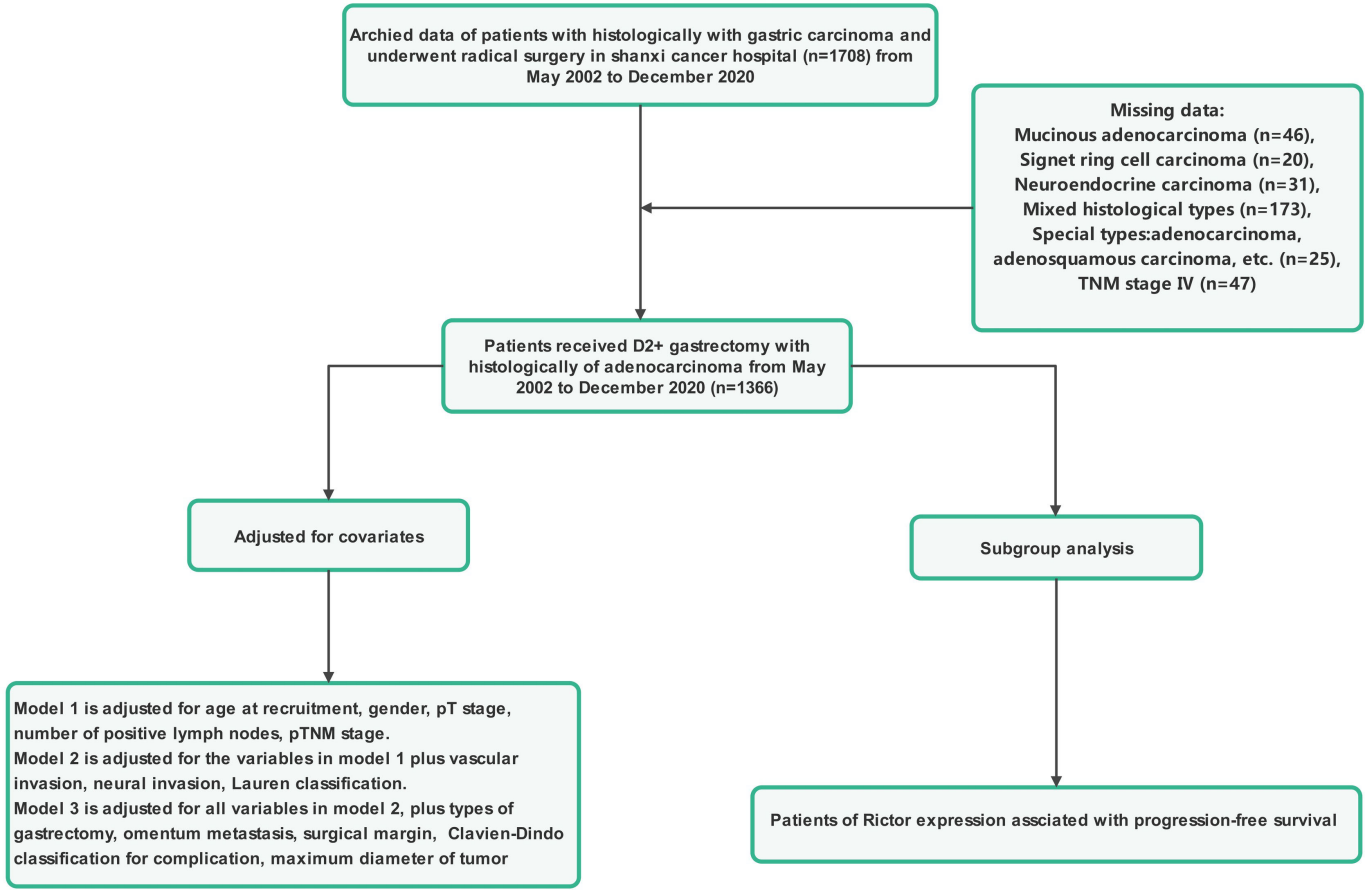
The flowchart outlining the research process.

Patients included in this study had to meet several criteria: they had to have histological confirmation of gastric adenocarcinoma, have complete clinicopathological and follow-up data, have no severe organ damage post-surgery, and have no other malignant tumors or causes of death unrelated to gastric cancer. Those who had other systemic tumors, incomplete clinical data, underwent palliative or bypass surgery, or were confirmed to have non-gastric cancer were excluded from the study. Tumor staging was determined based on the American Joint Committee on Cancer (AJCC) 8th TNM classification. The research protocol was approved by the Ethics Committee of Shanxi Cancer Hospital, and the study adhered to the principles of the Declaration of Helsinki. Informed consent was obtained from all patients involved, indicating they had voluntarily and informed consent to participate in the study. Patient data were anonymized and kept confidential. **Figures 1**, **2** provide a flowchart outlining the research process.

To be included in the study, patients had to meet specific criteria. These criteria included having a confirmed diagnosis of gastric cancer through histological examination and undergoing surgery with the intent of curing the disease (R0). The researchers analyzed various factors that affected the outcome of the surgery. These factors included gender, age at the time of surgery, presence of vascular and neural invasion, tumor stage (pT stage), number of positive lymph nodes, Lauren classification, maximum tumor diameter, type of gastrectomy performed, presence of omentum metastasis, surgical margin status, degree of complications based on the Clavien-Dindo classification, expression of specific biomarkers (AE1/AE3, CK20, CDX-2, SATB-2, SYN, CGA, CD56, MLH1, PMS2, Her-2, MSH2, and MSH6). Electronic medical records of hospital visits and communication with the oncologist were used to determine the follow-up time. The follow-up period started with the last hospital visit and ended with the previous contact with the surgeon. PFS was calculated as the time between surgery and either death or disease progression.

### Immunohistochemistry

Immunohistochemical staining was performed following a two-step protocol in a nature-inspired style. Microwave antigen retrieval was utilized to enhance the efficiency of the process. Tissue slides were then placed in a controlled environment at 4°C overnight, where they were incubated with the Rictor primary antibody (1:400). Following this step, the sections were exposed to a secondary antibody for 60 minutes at room temperature. We observed the development of the sections through a microscope and employed a DAB solution to aid in visualization. To create contrast, a counterstain with hematoxylin was applied. The immunoreactivity results were assessed by two independent investigators who were unaware of any clinical details about the patients. A semiquantitative grading system was employed to evaluate the staining results. This system involved scoring based on the proportion of stained cells and the staining intensity. When scoring for the proportion of stained cells, we assigned values as follows: no staining (0), less than 1/3 staining (1), 1/3 to 2/3 staining (2), and greater than 2/3 staining (3). For staining intensity, the values assigned were as follows: none (0), weak (1), medium (2), and strong (3). A final staining score was achieved by summing the values from both variables. Scores ranging from 0 to 2 were classified as unfavorable, while scores ranging from 3 to 6 were classified as positive.

### Statistical analysis

Continuous variables were presented as mean ± standard deviation, while categorical variables were elucidated using frequency and percentage. The Student’s t-test and non-parametric tests were employed to discern differences between groups in continuous variables. Concurrently, the Chi-square test was applied to assess differences in categorical variables. The training cohort was exclusively used for constructing the nomogram, whereas the validation cohort was explicitly employed to verify its performance. This study applied multivariate Cox regression to identify independent prognostic variables, with results reported as hazard ratios and corresponding 95% confidence intervals (CI) and P values. After the multivariate Cox regression analysis, factors exhibiting p-values below the threshold of 0.05 were incorporated into the construction of prognostic nomograms for 3-year and 5-year PFS. The nomograms were created by performing Cox regression analysis on various PFS-related parameters, and internal validation was evaluated through 10,000 repetitions. We employed various complementary methods to assess different aspects of model performance, including discriminative ability, model calibration, and clinical utility. We utilized Harrell’s concordance index (C-index) and the area under the receiver operating characteristic curve (AUC) to evaluate discriminative ability. AUC between 0.5 and 0.7 indicates poor discrimination, 0.7 and 0.9 suggests moderate performance, and greater than 0.9 indicates excellent performance. Model calibration was measured through calibration plots. Clinical utility was assessed through decision curve analysis (DCA) (10, 11). Furthermore, individuals were stratified into two risk groups—low and high—according to the scores derived from the nomogram. Subsequently, Kaplan-Meier curves were employed to predict the survival for each group. By delving into patients’ risk stratification and survival rates, it is possible to identify high-risk individuals better, laying the groundwork for personalized medicine and more effective health management. All statistical analyses were conducted using two-tailed tests, and statistical significance was defined as a P value less than 0.05. Data processing was performed using a variety of software packages, including R software (version 4.3.2) and SPSS 25.0.

## Results

### Essential characteristics of training cohort and validation cohort of Rictor (-) and Rictor (+)

In the training cohort, we included 486 gastric adenocarcinomas with Rictor (-) and Rictor (+), including 496 gastric adenocarcinomas; each variable was balanced between the training cohort and validation cohort for Rictor (-) and Rictor (+) (**Table 1**). To assess Rictor protein expression in these cancerous tissues, we employed Immunohistochemistry, revealing that 676 out of 1366 samples (49.5%) displayed Rictor immunoreactivity, specifically localized to the cytoplasm of the tumor cells, as depicted in the additional figure. Meanwhile, the expression of Rictor was classified as positive in 21.9% (112/512) samples besides the cancerous tissues. **Table 2** presented that Rictor expression is an independent predictor of gastric adenocarcinomas, and the effect remains in a fully adjusted model (model 3).

**Table 1:** Demographics of study population.

Variables	Rictor (-) (n=486) Training cohort	Rictor (-) (n=204) Validation cohort	P	Rictor (+) (n=496) Training cohort	Rictor (+) (n=180) Validation cohort	P
	Mean ± SD/No (%)	Mean ± SD/No (%)		Mean ± SD/No (%)	Mean ± SD/No (%)	
Gender			0.076			0.361
Male	398 (81.9%)	155 (76.0%)		392 (79.0%)	148 (82.2%)	
Female	88 (18.1%)	49 (24.0%)		104 (21.0%)	32 (17.8%)	
Age (years)	58.34 ± 9.90	59.20 ± 10.24	0.739	59.01 ± 9.86	59.14 ± 9.88	0.902
pT stage			0.1			0.352
T1	106 (21.8%)	28 (13.7%)		107 (21.6%)	35 (19.4%)	
T2	23 (4.7%)	9 (4.4%)		20 (4.0%)	7 (3.9%)	
T3	142 (29.2%)	65 (31.9%)		136 (27.4%)	46 (25.6%)	
T4	215 (44.2%)	102 (50.0%)		233 (47.0%)	92 (51.1%)	
Number of positive lymph nodes			0.237			0.481
0	191 (39.3%)	68 (33.3%)		165 (33.3%)	63 (35.0%)	
1-2	93 (20.2%)	36 (17.6%)		95 (19.2%)	37 (20.6%)	
3-6	58 (10.9%)	27 (13.2%)		84 (16.9%)	30 (16.7%)	
≥7	144 (29.6%)	73 (35.8%)		152 (30.6%)	50 (27.8%)	
Vascular invasion			0.663			0.976
Negative	228 (46.9%)	92 (45.1%)		241 (48.6%)	87 (48.3%)	
Positive	258 (53.1%)	112 (54.9%)		255 (51.4%)	93 (51.7%)	
Neural invasion			0.16			0.953
Negative	269 (55.3%)	101 (49.5%)		262 (52.8%)	90 (50.0%)	
Positive	217 (44.7%)	103 (50.5%)		234 (47.2%)	90 (50.0%)	
Lauren classification			0.232			0.516
Intestinal	202 (41.6%)	78 (38.2%)		227 (45.8%)	74 (41.1%)	
Diffuse	158 (32.5%)	80 (39.2%)		158 (31.9%)	62 (34.4%)	
Mixed	126 (25.9%)	46 (22.5%)		11 (22.4%)	44 (24.4%)	
Type of gastrectomy			0.54			0.363
Proximal	49 (10.1%)	15 (7.4%)		47 (9.5%)	22 (12.2%)	
Distal	157 (32.3%)	69 (33.8%)		166 (33.5%)	61 (33.9%)	
Total	278 (57.2%)	120 (58.8%)		283 (57.1%)	97 (53.9%)	
PPG	2 (0.4%)	0		0	0	
Omentum metastasis			0.052			0.770
Negative	468 (96.3%)	202 (99.0%)		486 (98%)	177 (98.3%)	
Positive	18 (3.7%)	2 (1.0%)		10 (2%)	3 (1.7%)	
Surgical margin			0.757			0.672
Negative	458 (94.2%)	191 (93.6%)		485 (97.8%)	175 (97.2%)	
Positive	28 (5.8%)	13 (6.4%)		11 (2.2%)	5 (2.8%)	
Her-2			0.084			0.727
Negative	315 (64.8%)	118 (57.8%)		294 (59.3%)	104 (57.8%)	
Positive	171 (35.2%)	86 (42.2%)		202 (40.7%)	76 (42.2%)	
Clavien-Dindo classification for complication			0.756			0.569
GradeI-II	442 (90.9%)	184 (90.2%)		419 (84.5%)	130 (72.2%)	
GradeIII-V	44 (9.1%)	20 (9.8%)		77 (15.5%)	50 (27.8%)	
Maximum diameter of tumor			0.814			0.123
<6cm	319 (65.6%)	132 (64.7%)		340 (68.5%)	112 (62.2%)	
≥6cm	167 (34.4%)	72 (35.3%)		156 (31.5%)	68 (37.8%)	
AE1/AE3			0.93			0.787
Negative	135 (27.8%)	56 (27.5%)		100 (20.2%)	38 (21.1%)	
Positive	351 (72.2%)	148 (72.5%)		396 (79.8%)	142 (78.9%)	
CK20			0.916			0.909
Negative	413 (85.0%)	174 (85.3%)		311 (62.7%)	112 (62.2%)	
Positive	73 (15.0%)	30 (14.7%)		185 (37.3%)	68 (37.8%)	
CDX-2			0.713			0.104
Negative	305 (62.8%)	131 (64.2%)		250 (50.4%)	78 (43.3%)	
Positive	181 (37.2%)	73 (35.8%)		246 (49.6%)	102 (56.7%)	
SATB-2			0.113			0.719
Negative	387 (79.6%)	173 (84.8%)		419 (84.5%)	150 (83.3%)	
Positive	99 (20.4%)	31 (15.2%)		77 (15.5%)	30 (16.7%)	
SYN			0.282			0.632
Negative	365 (75.1%)	161 (78.9%)		377 (76.0%)	140 (77.8%)	
Positive	121 (24.9%)	43 (21.1%)		119 (24.0%)	40 (22.2%)	
CGA			0.258			0.955
Negative	405 (83.3%)	177 (86.8%)		406 (81.9%)	147 (81.7%)	
Positive	81 (16.7%)	27 (13.2%)		90 (18.1%)	33 (18.3%)	
CD56			0.371			0.915
Negative	382 (78.6%)	154 (75.4%)		265 (53.4%)	97 (53.9%)	
Positive	104 (21.4%)	50 (24.6%)		231 (46.6%)	83 (46.1%)	
MLH1			0.122			0.492
Negative	85 (17.5%)	26 (12.7%)		62 (12.5%)	19 (10.6%)	
Positive	401 (82.5%)	178 (87.3%)		434 (87.5%)	161 (89.4%)	
PMS2			0.525			0.523
Negative	150 (30.9%)	68 (33.3%)		123 (24.8%)	49 (27.2%)	
Positive	336 (69.1%)	133 (66.7%)		373 (75.2%)	131 (72.8%)	
MSH2			0.635			0.114
Negative	108 (22.2%)	42 (20.6%)		34 (6.9%)	19 (10.6%)	
Positive	378 (77.8%)	162 (79.4%)		462 (93.1%)	161 (89.4%)	
MSH6			0.724			0.179
Negative	84 (17.3%)	33 (16.2%)		53 (10.7%)	26 (14.4%)	
Positive	402 (82.7%)	171 (83.8%)		443 (89.3%)	154 (85.6%)	

SD, standard deviation; No, number.

**Table 2:** Crude and adjusted hazard ratios estimate for Progression-free survival(PFS).

	^#^Model 1	^*^Model 2	^&^Model 3
Rictor	1.191 (1.102-1.402)	1.183 (1.004-1.393)	1.214 (1.030-1.432)
*P* for trend	0.036	0.045	0.021
AE1/AE3	1.066 (0.779-1.459)	1.058 (0.772-1.451)	1.052 (0.766-1.444)
*P* for trend	0.689	0.726	0.754
CK20	0.9 (0.756-1.072)	0.889 (0.746-1.059)	0.845 (0.706-1.01)
*P* for trend	0.237	0.187	0.064
CDX-2	0.984 (0.836-1.158)	0.889 (0.746-1.059)	0.974 (0.826-1.149)
*P* for trend	0.846	0.892	0.759
SATB-2	0.97 (0.756-1.072)	0.989 (0.839-1.165)	0.966 (0.782-1.194)
*P* for trend	0.772	0.761	0.751
SYN	1.155 (0.954-1.397)	1.158 (0.956-1.402)	1.156 (0.952-1.404)
*P* for trend	0.139	0.133	0.143
CGA	0.87 (0.681-1.111)	0.889 (0.696-1.137)	0.863 (0.673-1.106)
*P* for trend	0.266	0.35	0.244
CD56	0.87 (0.681-1.111)	0.904 (0.761-1.074)	0.878 (0.738-1.044)
*P* for trend	0.221	0.251	0.141
Ki67	1.006 (1.001-1.011)	1.005 (1.000-1.010)	1.004 (0.999-1.008)
*P* for trend	0.02	0.03	0.163

^#^Model 1 is adjusted for age at recruitment, gender, pT stage, number of positive lymph nodes, and pTNM stage.

^*^Model 2 is adjusted for the variables in model 1 plus vascular invasion, neural invasion, and Lauren classification.

^&^Model 3 is adjusted for all variables in model 2, plus types of gastrectomy, omentum metastasis, surgical margin, Clavien-Dindo classification for a complication, and maximum diameter of the tumor.

### Development and Validation of the prediction model of PFS of Rictor (-)

**Figure 3:**
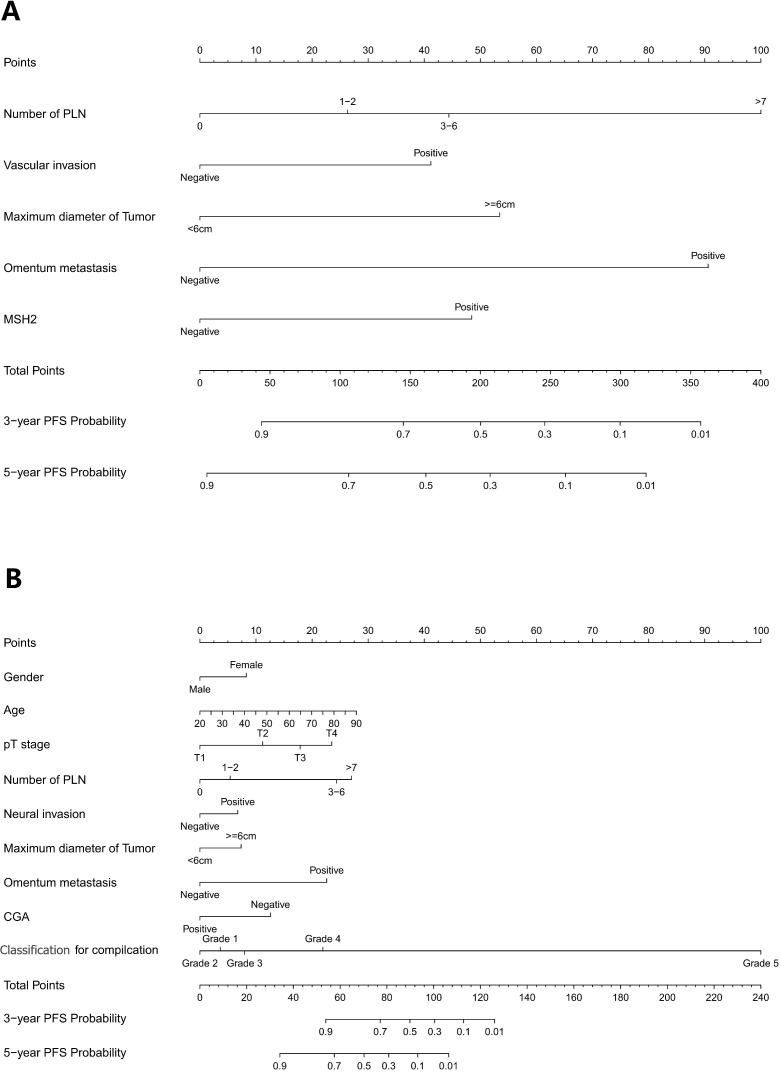
**(A)** Nomogram model to predict 3-year and 5-year PFS of Rictor (-) **(B)** Nomogram model to predict 3-year and 5-year PFS of Rictor (+).

The independent prognostic factors influencing progression-free survival (PFS) in patients with negative Rictor were investigated using multivariate Cox regression analysis. Results from **Table 3** revealed that significant factors were identified as the number of positive lymph nodes, vascular invasion, omentum metastasis, maximum tumor diameter, and MSH2 expression. These findings were obtained from the training cohort of 486 gastric cancer patients. By incorporating these variables into a nomogram model for Rictor (-), predicting the 3-year and 5-year PFS probabilities for these patients is possible. This nomogram model considers various factors that influence the likelihood of a favorable outcome, thus enabling effective prediction of a patient’s 3-year and 5-year PFS. **Figure 3** demonstrates the capability of the nomogram model to predict a favorable outcome for gastric cancer patients by considering the factors that affect 3-year and 5-year PFS. In the training cohort, the C-index was determined to be 0.760 (95%CI: 0.720-0.799), indicating a relatively reliable predictive capacity. Compared to the American Joint Committee on Cancer (AJCC) 8th edition TNM staging discrimination, the nomogram exhibited superior performance with a C-index of 0.683 (95%CI: 0.646-0.721).

**Table 3:** Multivariate analysis of PFS of training cohort of Rictor (-) and analyzed by Cox regression.

	B	SE	Wald	df	P	HR	HR (95%CI)
Number of positive lymph nodes			25.502	3	<0.001		
0 Vs 1-2	0.320	0.299	1.145	1	0.285	1.377	0.766-2.474
0 Vs 3-6	0.538	0.324	2.754	1	0.097	1.712	0.907-3.229
0 VS≥7	1.195	0.268	19.836	1	<0.001	3.302	1.952-5.586
Vascular invasion				1			
Negative Vs positive	0.491	0.221	4.948	1	0.026	1.633	1.060-2.517
Maximum diameter of Tumor				1			
<6cm Vs≥6cm	0.639	0.175	13.290	1	<0.001	1.894	1.344-2.671
Omentum metastasis				1			
Negative Vs Positive	1.044	0.293	12.689	1	<0.001	2.841	1.599-5.046
MSH2				1			
Negative Vs Positive	0.580	0.285	4.134	1	0.042	1.787	1.021-3.126

B, regression coefficient; SE, standard error; *df*, degree of freedom; HR, hazard ratio; CI, confidence interval.

**Figure 4:**
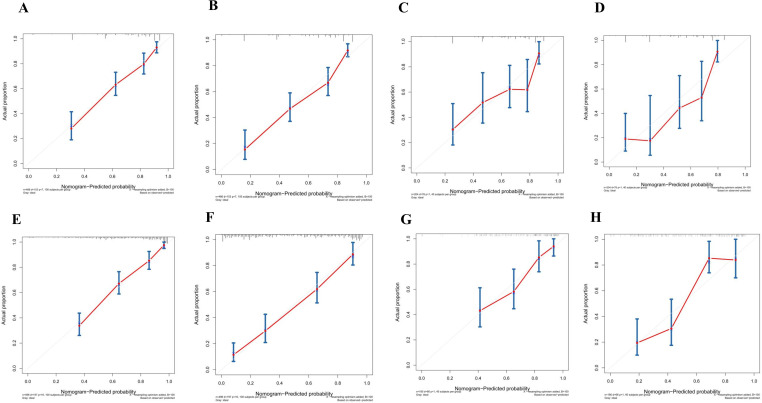
**(A)** Calibration curves of internal validation to predict 3-year PFS of Rictor (-) **(B)** Calibration curves of external validation to predict 3-year Rictor (-)**(C)** Calibration curves of internal validation to predict 5-year PFS of Rictor (-) **(D)** Calibration curves of external validation to predict 5-year PFS of Rictor (-) **(E)** Calibration curves of internal validation to predict 3-year PFS of Rictor (+) **(F)** Calibration curves of external validation to predict 3-year PFS of Rictor (+) **(G)** Calibration curves of internal validation to predict 5-year PFS of Rictor (+) **(H)** Calibration curves of external validation to predict 5-year PFS of Rictor (+).

**Figure 5:**
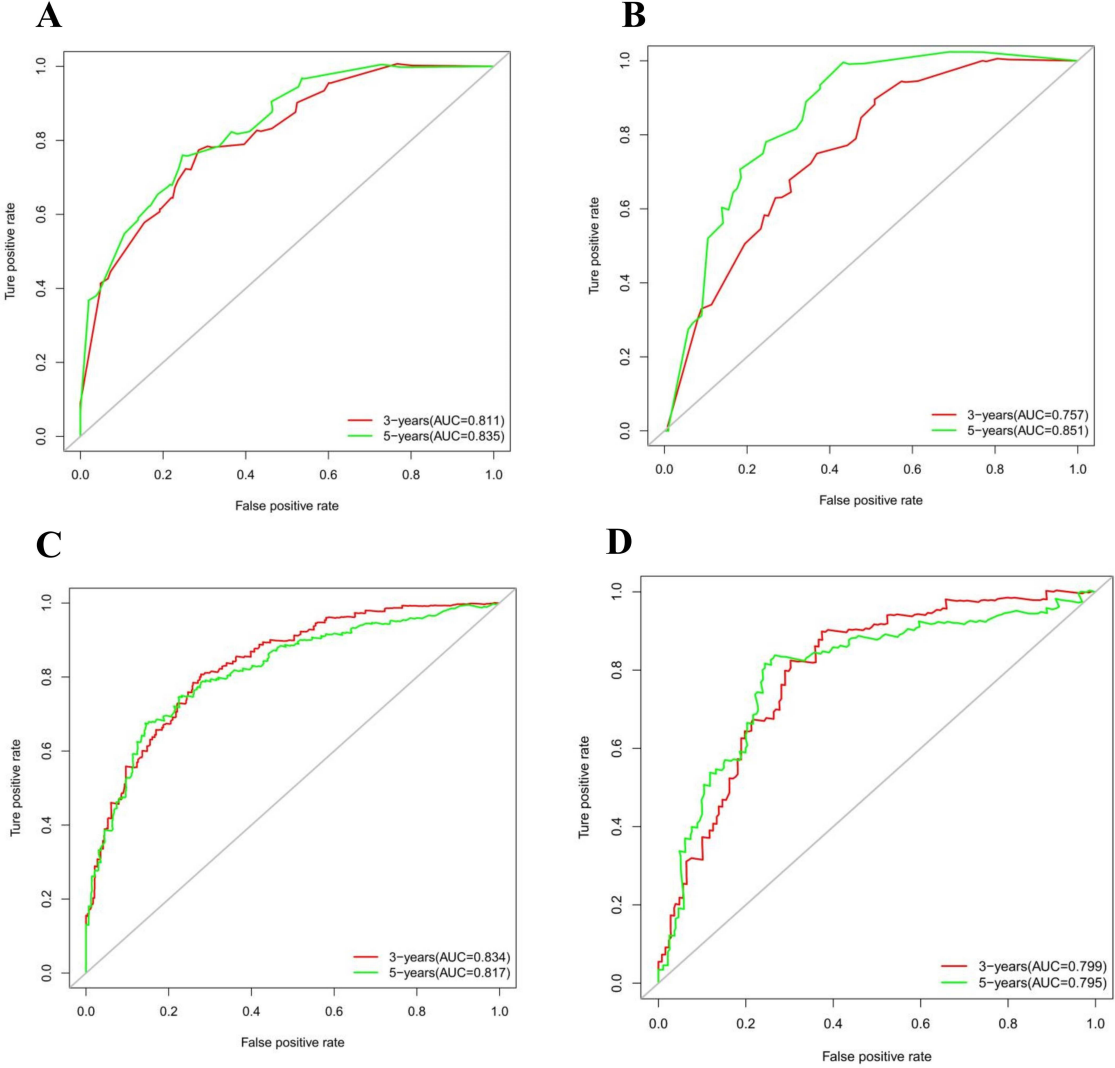
**(A)** Time-dependent receiver operating characteristic (t-ROC) curves of internal validation to predict PFS of Rictor (-) **(B)** Time-dependent receiver operating characteristic (t-ROC) curves of external validation to predict PFS of Rictor (-) **(C)** Time-dependent receiver operating characteristic (t-ROC) curves of internal validation to predict PFS of Rictor (+) **(D)** Time-dependent receiver operating characteristic (t-ROC) curves of external validation to predict PFS of Rictor (+).

**Figure 6:**
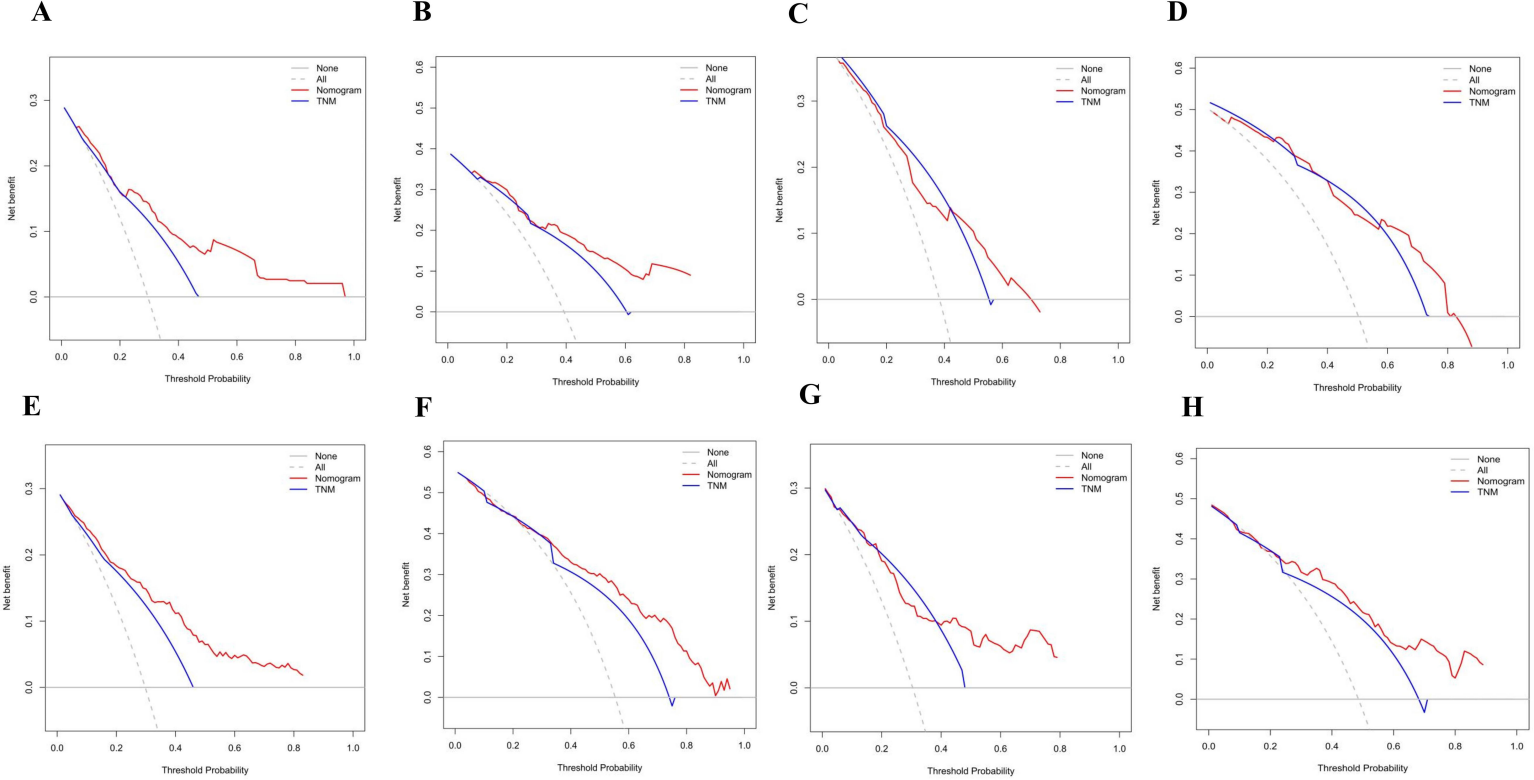
**(A)** Decision curve analysis (DCA) of internal validation to predict 3-year PFS of Rictor (-) **(B)** Decision curve analysis (DCA) of internal validation to predict 5-year PFS of Rictor (-) **(C)** Decision curve analysis (DCA) of external validation to predict 3-year PFS of Rictor (-) **(D)** Decision curve analysis (DCA) of external validation to predict 5-year PFS of Rictor (-) **(E)** Decision curve analysis (DCA) of internal validation to predict 3-year PFS of Rictor (+) **(F)** Decision curve analysis (DCA) of internal validation to predict 5-year PFS of Rictor (+) **(G)** Decision curve analysis (DCA) of external validation to predict 3-year PFS of Rictor (+) **(H)** Decision curve analysis (DCA) of external validation to predict 5-year PFS of Rictor (+).

The calibration curves in **Figures 4A–D** demonstrate that the predictions of PFS at 3 and 5 years using the nomogram are well-aligned with actual observations during internal and external validation. The internal validation, using time-dependent receiver operating characteristic (t-ROC) analysis, further confirms the favorable discriminative performance of the nomogram. The area under the curve (AUC) values for 3-year PFS and 5-year PFS are 0.811 (95% CI: 0.753-0.851) and 0.835 (95% CI: 0.776-0.878), respectively. Similarly, the external validation also shows promising results, with AUC values of 0.757 (95% CI: 0.699-0.853) for 3-year PFS and 0.851 (95% CI: 0.716-0.914) for 5-year PFS (**Figures 5A, B**). To assess the potential clinical benefit of the nomogram model, the DCA was conducted, comparing the predictions of 5-year and 3-year PFS between the AJCC 8th edition TNM staging and the nomogram. The internal validation C-index for the nomogram was 0.760 (95% CI: 0.720-0.799), surpassing the C-index of 0.683 (95% CI: 0.646-0.721) for the AJCC 8th edition TNM staging. Similarly, the external validation C-index for the nomogram was 0.736 (95% CI: 0.678-0.794), outperforming the C-index of 0.697 (95% CI: 0.653-0.742) for the AJCC 8th edition TNM staging. In both internal and external validation, the C-index values for the nomogram model were higher than those for the AJCC 8th edition TNM stage, indicating the superior predictive ability of the nomogram model (**Figures 6A–D**).

### Risk scoring of stratification system of PFS for Rictor (-)

**Figure 7:**
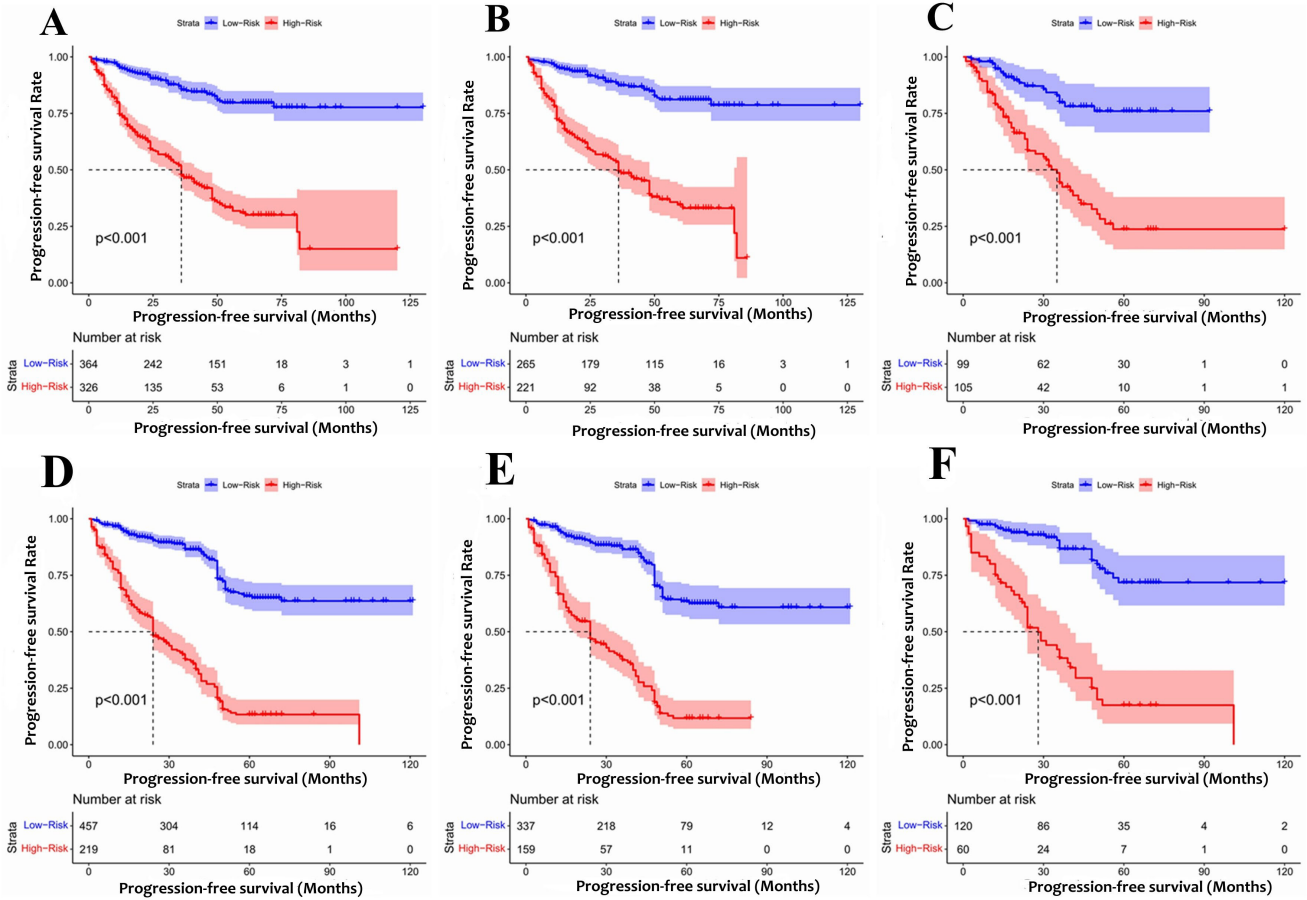
The Kaplan-Meier survival curves for patients with different scores **(A)** Rictor (-) of all cohort **(B)** Rictor (-) of training cohort **(C)** Rictor (-) of validation cohort **(D)** Rictor (+) of all cohort **(E)** Rictor (+) of training cohort **(F)** Rictor (+) of validation cohort.

**Figure 8:**
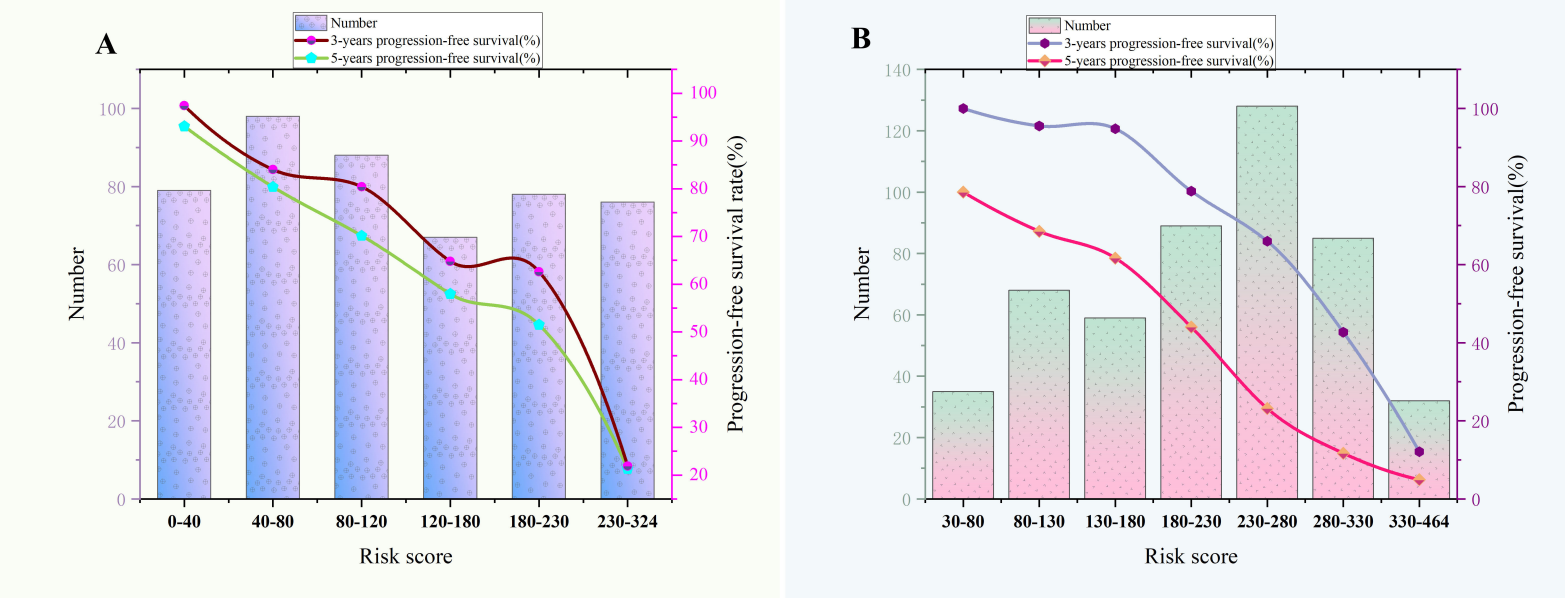
**(A)** The relationship between risk score and 3-year,5-year progression-free survival rate of Rictor (-). **(B)** The relationship between risk score and 3-year,5-year progression-free survival rate of Rictor (+).

According to the final nomogram model of Rictor (-), each patient was assigned a score and categorized. The X-tile software was utilized to determine the cutoff value for PFS scores in the training cohort, which included 486 patients. The log-rank test was then applied to compare survival times among different risk groups. The prognostic nomogram was used to calculate total scores. Upon setting a cutoff value of 122.34, the entire cohort, consisting of 690 individuals, was divided into two distinct groups with varying progression hazards, as depicted in **Figure 7**. The low-risk group (score ≤122.34) included 265 patients from the training sequence (n=486) and 99 patients from the validation sequence (n=204). On the other hand, the high-risk group (score >122.34) encompassed 221 patients from the training sequence (n=486) and 105 patients from the validation sequence (n=204). **Figure 7** illustrates the PFS curves for all three cohorts, demonstrating highly significant P values of less than 0.001. Notably, the median PFS for the low-risk group in the overall cohort (n=457) has not yet been reached, while it stands at 48 months for the high-risk group. In the training cohort (n=320), the median PFS for the low-risk group was 120 months, while the high-risk group had a median PFS of 36 months. Additionally, in the validation cohort (n=204), the median PFS for the low-risk group remains unknown, whereas the high-risk group had a median PFS of 35 months. The significant disparities in prognosis between the two risk groups further validate the exceptional performance of our model in stratifying risks. **Figure 8A** illustrates the correlation between risk score and progression-free survival rate, highlighting a noticeable decrease in the 5-year progression-free survival rate as the risk score escalates, especially evident above 120 (120-180: 52.5%, 180-220: 44.6%, 230-324: 7.7%). Similarly, the 3-year progression survival rate significantly drops once the risk score surpasses 180 (180-230: 62.6%, 230-324: 21.9%). These findings concisely represent the connection between risk scores and survival rates, aligning seamlessly with the risk stratification system for progression-free survival.

### Development and validation of the prediction model of PFS of Rictor (+)

**Table 4** presents the findings from the multivariate Cox regression analysis conducted in the Rictor (+) training cohort of 496 patients, highlighting the independent risk factors influencing progression-free survival (PFS). These risk factors encompass gender, age, pT stage, number of positive lymph nodes, maximum tumor diameter, neural invasion, omentum metastasis, Clavien-Dindo classification for complications, and expression of CGA. Utilizing these nine variables, the nomogram can estimate the likelihood of 3-year and 5-year PFS in gastric cancer patients with Rictor positivity. This tool is valuable in identifying patients more likely to achieve favorable outcomes. **Figure 3B** showcases the nomogram model, incorporating the aforementioned independent predictors, to predict 3-year and 5-year PFS. The C-index for predicting PFS in the training cohort is 0.755 (95%CI: 0.728-0.782). When compared to the discriminatory ability of the AJCC 8th edition TNM staging, the nomogram demonstrates superior performance with a higher C-index of 0.712 (95%CI: 0.688-0.737).

**Table 4:** Multivariate analysis of PFS of training cohort of Rictor (+) and analyzed by Cox regression.

	B	SE	Wald	df	P	HR	HR (95%CI)
Gender							
Male Vs Female	0.525	0.170	9.520	1	0.002	1.690	1.211-2.359
Age	0.021	0.008	7.575	1	0.006	1.021	1.006-1.036
pT stage			7.416	3	0.060		
T1 Vs T2	0.698	0.660	1.117	1	0.290	2.010	0.551-7.335
T1 Vs T3	0.909	0.471	3.732	1	0.053	2.483	0.987-6.248
T1 Vs T4	1.197	0.475	6.340	1	0.012	3.309	1.304-8.400
Number of positive lymph nodes			40.193	3	<0.001		
0 Vs 1-2	0.344	0.319	1.165	1	0.280	1.410	0.755-2.633
0 Vs 3-6	1.308	0.305	18.410	1	<0.001	3.700	2.035-6.727
0 VS≥7	1.424	0.292	23.767	1	<0.001	4.153	2.343-7.360
Neural invasion				1			
Negative Vs positive	0.360	0.176	4.167	1	0.041	1.433	1.014-2.024
Maximum diameter of Tumor				1			
<6cm Vs ≥6cm	0.372	0.155	5.757	1	0.016	1.450	1.070-1.965
Omentum metastasis				1			
Negative Vs Positive	1.040	0.372	7.803	1	0.005	2.830	1.364-5.872
Clavien-Dindo classification for complication				1			
Grade I-II Vs Grade III-V	0.183	0.091	4.023	1	0.045	1.200	1.004-1.435
CGA				1			
Negative Vs Positive	-0.620	0.295	4.428	1	0.035	0.538	0.302-0.958

B, regression coefficient; SE, standard error; *df*, degree of freedom; HR, hazard ratio; CI, confidence interval.

The results in **Figures 4E–H** exhibit the close alignment of the calibration curves from internal and external validations with the actual observations. This confirms the consistency of the nomogram’s predictions. A time-dependent ROC curve was generated further to evaluate the accuracy of the nomogram’s predictions. AUC for t-ROC was calculated for the 3-year and 5-year PFS models. The internal validation AUC for the 3-year PFS was 0.834 (95%CI: 0.746-0.823), while the external validation AUC was 0.799 (95%CI: 0.699-0.868). For the 5-year PFS, the internal validation AUC was 0.769 (95%CI: 0.718-0.821), and the external validation AUC was 0.795 (95%CI: 0.675-0.895). The AUC values exceeded expectations for internal and external validations, indicating the exceptional performance of the model (**Figures 5C, D**).

The application of DCA revealed that our nomogram (**Figures 6E–H**) delivered clinical benefits. Furthermore, the DCA analysis demonstrated that our nomogram outperformed the AJCC TNM classification. Our nomogram showed a higher net benefit in both the training and validation cohorts compared to the AJCC TNM staging. The internal validation C-index was 0.795 (95% CI: 0.764-0.825), surpassing the C-index of the AJCC 8th edition TNM staging, which was 0.693 (95% CI: 0.662-0.723). External validation also supported our findings, with a C-index of 0.769 (95% CI: 0.718-0.821) for our nomogram compared to the AJCC 8th edition TNM staging with a C-index of 0.715 (95% CI: 0.669-0.760).

### Risk scoring of stratification system of PFS for Rictor (+)

Based on the final nomogram model, each patient’s score is calculated—our cutoff value for PFS of the training cohort (n=496) generated by the X-tile software. The log-rank test method compared survival times among the different risk groups. Total scores were calculated according to the prognostic nomogram. According to the cutoff value of 265.08, the entire cohort (n=676) was divided into two groups with totally different disease progression risk probabilities (**Figures 7D–F**): the low-risk group [0 ≤ 265.08, including 337 patients in the training cohort (n=496) and 60 patients in the validation cohort (n=180)], and the high-risk group [>265.08, including 159 patients in the training cohort (n=496) and 60 patients in the validation cohort (n=180)]. **Figures 7D–F** shows progression-free survival curves stratified by risk scores for all cohorts, training cohorts, and validation cohorts, with P values less than 0.001 for all three cohorts. The median PFS of the low-risk group in the entire cohort (n=676), training cohort (n=496), and validation cohort (n=180) has not been reached, and the median PFS of the high-risk group of three cohorts were 24 24, 28 months, respectively. Statistical differences in prognosis between the two risk stratification groups further indicated that our model has good risk stratification performance. **Figure 8B** elegantly captures the relationship between risk score and progression-free survival rate, showcasing a clear downward trend in 3-year survival rates as risk scores increase. The graph depicts a sharp decline in survival rates once the score exceeds 230, with rates plummeting to 66% in the 230-280 range, 42.7% in the 280-330 range, and a mere 12.1% in the 330-364 range. Similarly, the 5-year survival rate shows a significant drop after surpassing a score of 230, declining from 56% in the 230-280 range to a mere 6.25% in the 330-364 range. These findings visually highlight the strong correlation between risk score and survival outcomes, aligning with the risk stratification system used in this study. It emphasizes the critical connection between risk assessment and survival prognosis within the research context.

## Discussion

Our study utilized a blend of clinical features, pathological parameters, and tumor molecular markers to identify key variables using COX regression analysis. Our main goal was to construct a nature-inspired forest plot for predicting patients’ PFS based on Rictor expression. The intricate forest plot for Rictor-positive individuals includes nine significant variables: gender, age, pT stage, number of positive lymph nodes, nerve invasion, tumor maximum diameter, serosal invasion, Clavien-Dindo postoperative complication grade, and CGA expression. Conversely, the prediction system for Rictor-negative individuals incorporates factors such as the number of positive lymph nodes, vascular invasion, omentum metastasis, maximum tumor diameter, and MSH2 expression. Acknowledging that various factors may impact patient outcomes post-radical gastrectomy at different medical centers, we undertook internal and external validations to evaluate our model’s performance thoroughly. The validations illustrated that our model demonstrated favorable predictive accuracy, calibration, discrimination, and clinical utility. Furthermore, we conducted a population-based analysis and divided patients into two risk groups to enhance the accuracy of the forest plot and create a stratified prognostic model. Our holistic approach offers clinical practitioners valuable guidance and improves communication between patients and healthcare providers. By integrating multiple factors and conducting extensive validations, our model can aid physicians in predicting patients’ PFS based on Rictor expression. This information can facilitate informed treatment decisions and tailor personalized treatment strategies for patients.

The Rictor protein is a vital component of the rapamycin-insensitive complex mTORC2, acting as a scaffolding protein. Its interaction with mTOR is crucial for activating mTOR’s kinase activity (12). With a molecular weight of 192 kDa, Rictor shares homology with the AVO3 protein found in the TOR2 complex of yeast. While the specific functions of different structural domains within the Rictor molecule remain uncertain, its conservation in eukaryotes is relatively well-preserved. With a total of 1708 amino acid residues, the precise roles of these structural domains are not yet fully understood. Notably, although Rictor and other related proteins have a conserved region spanning roughly 200 amino acid residues, the long C-terminal extension at the carboxyl end of the molecule is distinct from those found in other proteins (13).

Previous studies have provided some insights regarding the correlation between Rictor expression and gastric adenocarcinoma prognosis, but further research is needed to understand its impact fully (9, 14, 15). Bian et al. found that Rictor expression was not a standalone prognostic indicator when other factors were considered (13). On the other hand, Cao et al. demonstrated that Rictor plays a role in inhibiting tumor apoptosis and activating Cav 1 through the Akt signaling pathway, leading to a worse prognosis in gastric cancer (16). Additionally, Rictor amplification has been identified as a rare genomic alteration with therapeutic implications in gastric cancer (15). Lang et al. discovered that down-regulating Rictor can be mediated through mTORC2-induced Akt activation in gastric and pancreatic cancer cells (17). Wang et al. reported a high positive rate of Rictor in gastric cancer tissues and linked its upregulation to a poor prognosis in patients (8). In our study, we conducted a retrospective analysis of gastric cancer patients and determined Rictor to be an independent adverse prognostic factor in progression-free survival (18). With a comprehensive long-term follow-up, we aimed to shed light on the significance of Rictor in predicting outcomes for gastric adenocarcinoma patients. We constructed predictive models for predicting survivals based on different Rictor statuses.

Stratifying the survival risk of gastric cancer patients in a nature-inspired approach allows us to distinguish between low-risk and high-risk groups by establishing specific cut-off values. These values are determined through the analysis of ROC curves. The optimal threshold is near the top-left corner, where the balance between sensitivity and false positive rate is most advantageous. Zheng et al. (19) utilized preoperative blood indicators to predict post-radical gastrectomy survival in gastric cancer patients and to categorize risks based on their discoveries. In another study, a nomogram was developed using the Surveillance, Epidemiology, and End Results (SEER) database to anticipate the overall survival of patients with gastric cancer that has spread to the lungs (20). Advanced imaging techniques such as MRI and CT-based radiomics have also been explored to forecast gastric cancer survival (21, 22). Furthermore, genomic studies have identified genes linked to gastric cancer survival and have developed nomograms to refine risk stratification for these patients (23). In contrast to previous research, our work integrates proteomics, clinical characteristics, and pathological parameters into our models, enhancing risk stratification through cut-off values.

The essential role of MSH2 in human DNA repair mechanisms has been firmly established, and its functional significance in the context of gastric cancer is increasingly recognized (24, 25). This protein is a key component of the mismatch repair (MMR) system and is pivotal in maintaining genomic stability. Dysfunction of MMR genes, including MSH2, is associated with hereditary forms of gastric cancer, such as Lynch syndrome—the most prevalent genetic predisposition to this malignancy. Mutations in MMR genes can lead to the development of hereditary gastric cancer, with specific genetic alterations influencing the risk profile (26, 27). The present study investigates the correlation between MSH2 expression levels and the PFS of patients with primary gastric adenocarcinoma. We identified a significant role for MSH2 in predicting clinical outcomes, suggesting that this protein may serve as a prognostic biomarker. Notably, the interplay between MSH2 and Rictor, a protein that regulates mTORC2, appears to be a promising avenue for further exploration. Understanding the molecular crosstalk between these factors could potentially yield novel insights into the pathogenesis and treatment of gastric cancer.

CGA, a 439-KD protein, resides within the secretory granules of numerous neuroendocrine cells, both benign and malignant, and is pivotal in the regulation of protein storage and secretion processes (28). This protein has been proposed as a biomarker for the detection of neuroendocrine neoplasms (29, 30), and its utility as a diagnostic tool for gastric cancer, using microarray and tissue array technologies, has been suggested (31). In the context of pancreatic neuroendocrine tumors, CGA expression is commonly detected, with a trend indicating that tumors with metastases exhibit reduced CGA protein levels compared to those confined to the primary site (32). Despite this, research into CGA’s role in gastric cancer has been limited, primarily confirming its presence in neuroendocrine differentiated entities within diffuse gastric carcinomas (33, 34). The current study identifies CGA expression as a prognostic factor for patients with primary gastric adenocarcinoma, particularly in the context of Rictor (+) status, which is associated with PFS. These findings suggest a significant involvement of CGA in the pathogenesis of gastric adenocarcinoma. Furthermore, it underscores the necessity for additional research to corroborate the interplay between CGA and Rictor in the development and prognosis of gastric adenocarcinoma.

We have developed a predictive and risk stratification model using a prognosis score plot to distinguish different risk levels among patients with negative and positive Rictor protein. This model allows us to identify low-risk patients who can forego additional postoperative treatment. On the other hand, high-risk patients should consider targeted therapies that specifically target Rictor-positive indicators and multiple therapies for Rictor-negative indicators.

Despite promising results, the current study has several limitations that should be acknowledged. Firstly, the model construction and validation were based on training and validation cohorts from a single center; therefore, it is necessary to validate the findings further using data from other medical centers. Secondly, there needs to be more consistency between the effectiveness of the line plot model in predicting 5-year PFS and the actual data, which warrants further investigation. Thirdly, the study did not distinguish between patients with early and late-stage gastric adenocarcinoma, and the predictive performance may vary among patients with different stages of gastric adenocarcinoma. Lastly, it is important to note that all tumor markers explored in this research - including AE1/AE3, CK20, CDX-2, SATB-2, SYN, CGA, CD56, MLH1, PMS2, Her-2, MSH2, MSH6, and Rictor - were only subjected to qualitative analysis, lacking quantitative analysis.

In conclusion, the increasing importance of Rictor, a recent addition to the mTORC2 team, must be considered in recent times. Rictor is crucial in cell proliferation, growth, and differentiation processes (35). However, there are still many unanswered questions about how it operates. Further investigation into Rictor will help us understand cellular activity regulation better and provide new perspectives and strategies for treating tumors and related diseases. This, in turn, presents potentially revolutionary drug targets.

## Conclusion

This is the first reported risk stratification for Rictor expression in gastric carcinoma. Our model identifies low-risk patients who may not require additional postoperative treatment. Conversely, high-risk patients should consider targeted therapies that specifically target Rictor-positive indicators.

## Data Availability

The datasets presented in this study can be found in online repositories. The names of the repository/repositories and accession number(s) can be found in the article/supplementary material.
